# Biofilm Formation on Three High-Performance Polymeric CAD/CAM Composites: An In Vitro Study

**DOI:** 10.3390/polym17050676

**Published:** 2025-03-03

**Authors:** Sarah Almuhayya, Reema Alshahrani, Rehaf Alsania, Alhanoof Albassam, Hammad Alnemari, Rua Babaier

**Affiliations:** 1 Department of Clinical Laboratory Science, College of Applied Medical Sciences, King Saud University, P.O. Box 10219, Riyadh 12372, Saudi Arabia; 2 College of Dentistry, King Saud University, P.O. Box 60169, Riyadh 11545, Saudi Arabia; 3 Microbiology Laboratory, College of Dentistry, King Saud University, P.O. Box 60169, Riyadh 11545, Saudi Arabia; halnemari@ksu.edu.sa; 4 Department of Prosthetic Dental Sciences, College of Dentistry, King Saud University, P.O. Box 60169, Riyadh 11545, Saudi Arabia

**Keywords:** CAD/CAM, fiber-reinforced composites, high-performance polymers, PEEK, *Streptococcus mutans*, biofilm adhesion, wettability, surface roughness, confocal laser scanning microscopy, scanning electron microscopy

## Abstract

Reinforced polymeric materials are investigated as novel non-metal alternatives for prosthetic frameworks. This study examined the adherence of *Streptococcus mutans* to three *high-performance polymeric* (HPP) composites focusing on their microstructural composition, wettability, and surface roughness. Three CAD/CAM HPP composites [two fiber-reinforced composites, CarboCad (CC) and TRINIA (TR), and one ceramic-reinforced polyether ether ketone, DentoPEEK (PK)], were sectioned into ten beam- and ten plate-shaped specimens from each material. Surface properties (n = 10) were analyzed by water wettability and roughness measurements (Ra and Rz). The biofilm adherence was determined by calculating the number of *S. mutans* through colony-forming units (CFUs). Representative images were obtained using a confocal laser scanning microscope (CLSM) and scanning electron microscopy (SEM). The data were analyzed using Welch one-way ANOVA and Dunnett T3 post hoc tests. The results showed significant differences in roughness (Ra) across the materials, ranked from highest to lowest as follows: TR, 0.231 µm; CC, 0.194 µm; and PK, 0.161 µm (*p* = 0.0001). The contact angle averages varied from 51.36° to 91.03°, with PK exhibiting the highest wettability (*p* = 0.0012). However, *S. mutans* adherence was markedly reduced in PK (1.96 CFU/mm^2^, *p* = 0.0001) in comparison to TR and CC (2.86 and 2.98 CFU/mm^2^, respectively). Consequently, the fiber-reinforced composites (CC and TR), despite their low wettability, exhibited greater susceptibility for bacterial adherence than the smoother and more wettable PK, highlighting the substantial impact of their surface roughness and microstructural variability.

## 1. Introduction

All soft and hard surfaces within the oral cavity are coated by the acquired pellicle, which is subsequently followed by bacterial colonization. Biofilms are structured microbial communities embedded in a self-produced extracellular polymeric matrix that shields them from environmental stresses, antimicrobial substances, and host immunological reactions [1]. These communities exhibit unique characteristics such as increased antibiotic resistance, modified metabolic activity, and enhanced ability to persist in challenging conditions [2]. Their formation involves initial bacterial adhesion, microcolony proliferation, and maturity, resulting in a robust structure that is challenging to eliminate [3].

Biofilms are clinically significant due to their role in chronic infections, including those affecting the oral cavity, contributing to dental caries, periodontitis, implant-related infections, and denture-associated stomatitis [4]. The susceptibility of dental restorative material to bacterial adhesion is crucial for their clinical longevity [5,6]. The adherence of numerous pathogenic bacteria in a complex and dynamic oral environment poses challenges, requiring a more profound comprehension of the interaction between these newly developed materials and bacterial adhesion. *Streptococcus mutans* (*S. mutans*), a frequently investigated bacterium in dental material research [7], is pivotal in dental plaque production, hence influencing the durability and efficacy of restorative treatments [8]. Dental materials are designed to endure environmental fluctuations in the oral cavity, such as thermal, chemical, mechanical, and biological changes.

Nevertheless, microbial adhesion is substantially influenced by surface wettability and microstructural composition [9]. Furthermore, increased surface roughness may result in greater plaque accumulation, hence increasing the risk of periodontal inflammation [10]. Recent studies indicate that surface roughness and hydrophobicity significantly influence the biofilm formation of 3D-printed resins in comparison to conventional resins [11] and conventionally fabricated zirconia and titanium [12].

The relationship between wettability and biofilm adhesion on dental materials is extensively established, exhibiting a robust positive correlation [11,13]. Surfaces exhibiting increased contact angles, denoting greater hydrophobicity, generally lead to reduced biofilm formation, including that of *S. mutans* and *Staphylococcus aureus*. The lower wettability can be attributed to the water-repellent characteristics of hydrophobic surfaces, which inhibit bacterial colonization [14,15]. Conversely, reduced contact angles (increased wettability) associated with more hydrophilic surfaces promote bacterial adhesion, as these surfaces attract moisture, creating conditions conducive to biofilm development [16,17].

Advanced material technologies and computer-aided design and computer-aided manufacturing (CAD/CAM) systems have produced framework materials with superior physical and mechanical qualities. Recently, *high-performance polymer* (HPP) composites have gained attention due to their exceptional *strength-to-weight ratio* and potential for improved biocompatibility in dental applications [18,19]. HPP composites are proposed as a viable alternative for conventional metals and metal oxides in implant-supported frameworks and prosthetics [19,20,21]. Modifying these polymer-based composites has improved their microstructural compositions and mechanical characteristics, broadening their clinical applications. Microstructure reinforcement could be achieved by incorporating various types of glass or carbon fibers, known as fiber-reinforced composites (FRCs), or by combining ceramic fillers with various matrix compositions.

A few studies have examined three new commercially available HPP products: two comprising epoxy resin reinforced with either layered glass fibers or a random network of carbon fibers [22,23,24] and the third consisting of polyether ether ketone (PEEK) filled with fine ceramic particles [22,23]. Research showed that their distinctive microstructural variations influenced their surface and mechanical properties during aging in food-simulating liquids [20,21,24]. The results found that the reinforced PEEK exhibited more stability than the two FRCs for surface and mechanical properties.

Nevertheless, no comparable studies examined their susceptibility to bacterial adhesion. Therefore, this study investigated the adherence of *S. mutans* to three *high-performance polymeric* (HPP) CAD/CAM composites by examining their surface characteristics, roughness, and wettability. The null hypotheses are as follows:

1. No differences in surface roughness or wettability exist among the three HPP composites.
2. No differences in the biofilm adhesion ability of *S. mutans* on the three HPP composites.

## 2. Materials and Methods

### 2.1. Specimen Preparation

Three high-performance polymeric CAD/CAM composite blocks were investigated (Table 1): two fiber-reinforced composites (FRCs) [CarboCAD (CC) and TRINIA (TR)] and a particle-filled polyether ether ketone (PK). Twenty specimens were sectioned from each material into plates (n = 10) and beams (n = 10) with a diamond disc saw (IsoMet 1000 Precision saw, Buhler, Lake Bluff, IL, USA). Every surface of the specimens was manually polished using SiC grinding papers (Buhler, Lake Bluff, IL, USA) with grits P600 and P800 to smooth any sharp edges. Specimen dimensions were measured using a digital micrometer (AO+, Ellenburg depot, NY, USA) for plates (14 mm × 12 mm × 2 mm) and beams (8 mm × 4 mm × 2 mm) followed by ultrasonic cleaning for five minutes. CC beams were sintered at 80 °C for 2 h in accordance with the manufacturer’s specifications, whereas TR and PK required no firing. All specimens were designated with a code and serial number to guarantee consistent and accurate repeated measurements.

### 2.2. Surface Roughness

The roughness of ten plates from each material was determined by employing advanced non-contact equipment (Talysurf CLI 1000, Taylor Hobson Precision, Leicester, UK). A white light profilometer scanned small areas (1 mm^2^) of the specimens with a high resolution of 1001 points at a speed of 500 µm/s, acquiring detailed data through three scans organized in a grid configuration for consistency. Key two-dimensional roughness parameters were calculated in µm, including Ra (average surface roughness) and Rz (the vertical disparity between the highest peak and the lowest valley), as delineated by ISO 25178/2017 [25].

### 2.3. Wettability (Contact Angle Measurements)

Contact angle measurements were obtained for the same ten plates from each material specimen using the sessile drop method with a calibrated Goniometer (Ramé-Hart 100-FO, Netcong, NJ, USA), in accordance with ASTM D7334/08 [26]. The angles of a 2.0 µL distilled water droplet on each specimen were measured after 20 s using DROPimage, Version 2.5.01 software. The mean readings (mean ± SD) were recorded in degrees and repeated thrice for each specimen.

### 2.4. Scanning Electron Microscopy (SEM)

To illustrate the surface characteristics of each HPP composite, one representative plate (n = 1) was chosen for SEM imaging at baseline (prior to biofilm adherence). Each specimen was coated with gold, and images were captured in backscattered electron mode at 15 kV at a magnification of 250× using SEM (JSM-6610 LV, JEOL Co., Tokyo, Japan).

### 2.5. Bacterial Cell Growth Conditions

The *S. mutans* strain ATCC 25,175 was acquired from the culture collection at the microbiology laboratory at King Saud University. Subsequently, *S. mutans* were cultivated on brain heart infusion (BHI) agar (Difco, Detroit, MI, USA) and incubated for 24 h at 37 °C in a 10% CO_2_ chamber. Bacterial colonies were subsequently suspended in phosphate-buffered saline (PBS; Sigma-Aldrich, Darmstadt, Germany). The suspension was subsequently adjusted to 0.5 McFarland, equating to a microbial concentration of roughly 1.5 × 10^8^ cells/mL.

#### 2.5.1. In Vitro Biofilm Adhesion Assay

**Figure 1:**
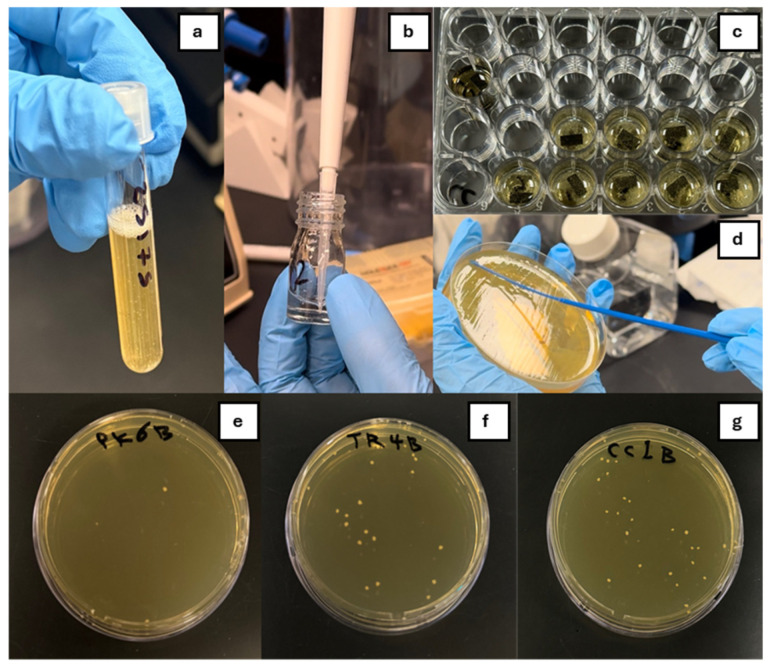
Experimental process of bacterial adhesion and colony formation on beam specimens. (**a**) *S. mutans* culture suspension preparation; (**b**) dilution of bacterial suspension; (**c**) representative CC specimens placed in sterile 24-well polystyrene tissue culture plate; (**d**) spread of 0.1 mL aliquots from each tube onto BHI agar plates; (**e**–**g**) *S. mutans* colonies on BHI agar (PK, TR, CC).

The sterilized beam specimens (autoclaved at 121 °C for 15 min at 15 psi) were positioned in individual sterile Petri dishes, each containing 5 mL of sterile artificial saliva (Sigma-Aldrich Chemie GmbH, Deisenhofen, Germany). The beams were subsequently incubated at 37 °C in a 10% CO_2_ atmosphere for 1 h to develop the pellicle layer. Subsequently, sterilized specimens (n = 7) were positioned in a sterile 24-well polystyrene tissue culture plate, with each specimen allocated to an individual well. Afterwards, 2 mL of the produced bacterial suspension was applied to the surface of each specimen and incubated for 24 h at 37 °C in a CO_2_ chamber with agitation at 120 rpm. Specimens were subsequently extracted from each well and dip rinsed in sterile PBS twice, and then adhered bacterial cells were detached using vortex mixing. These were then transferred into separate 10 mL tubes containing 1.5 mL PBS and sonicated for 30 s to disperse the adherent bacteria. The specimens were subsequently extracted from the suspension and subjected to repeated dilution. Subsequently, 0.1 mL aliquots from each tube were inoculated onto BHI agar plates and incubated for 48 h at 37 °C in a CO_2_ atmosphere, as illustrated in Figure 1. The colonies of *S. mutans* were visually counted using a Reichert Quebec^®^ Darkfield Colony Counter (Cambridge Instruments, Depew, NY, USA), and the average colony-forming units (CFUs) were calculated (CFU/mm^2^). This protocol adhered to that of a prior study [27].

#### 2.5.2. Surface Analysis Confocal Laser

To study the biofilm surface quality and pattern of distribution, two randomly selected beams from each material were examined using a confocal laser scanning microscope (CLSM) (Nikon C2 confocal laser microscope, Nikon Instruments Inc., Melville, NY, USA). Selected specimens were gently extracted from the wells, rinsed twice with sterile PBS to exclude non-adherent cells, and stained using the fluorescent LIVE/DEAD BacLight Bacterial Viability dye (Molecular Probes, Eugene, OR, USA). The mounted specimens were photographed with the CLSM with a 10/0.49 NA air immersion objective. The four corners of each beam were scanned. The fluorescence signal of live bacteria was acquired by illuminating the specimens with a 488 nm laser, and the emission was captured using a 520–540 nm bandpass filter (green channel). The fluorescence of dead bacteria was detected using excitation from a 568 nm laser and emission through a 600–630 nm filter (red channel).

### 2.6. Statistical Analysis

The sample size was established based on prior research concerning roughness [20], contact angle measurements, and colony-forming units [27]. Data were analyzed statistically using SPSS 23.0 (IBM SPSS Statistics, SPSS Inc., New York, NY, USA). The Shapiro–Wilk test proved normality; however, the Levene test indicated a lack of homogeneity of variance. Consequently, bivariate analysis was performed employing Welch one-way analysis of variance (ANOVA) and Dunnett T3 post hoc testing to compare the means of contact angle, roughness (Ra and Rz), and Log_10_ of CFUs among the three materials examined. Spearman’s correlation coefficient was employed to examine correlations among the measurements. A *p*-value of <0.05 and 95% confidence intervals were used to indicate statistical significance and the precision of the results.

## 3. Results

**Figure 2:**
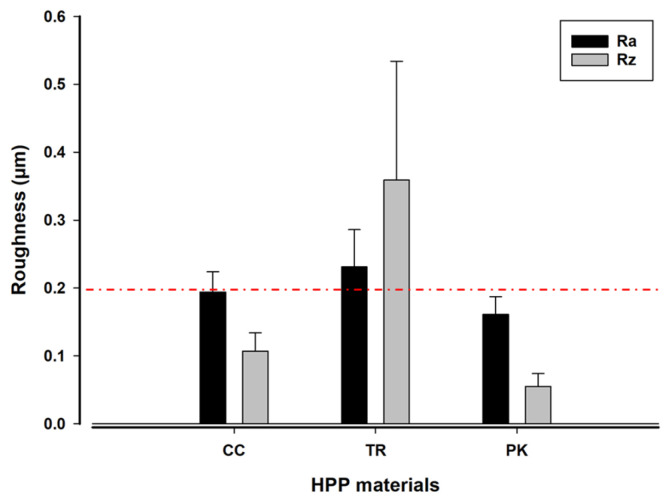
Roughness measurements (Ra and Rz) of three HPP composites (CC, TR, and PK). *The dashed red line marks the maximum clinically accepted threshold for roughness* (Ra = 0.2 µm).

**Figure 3:**
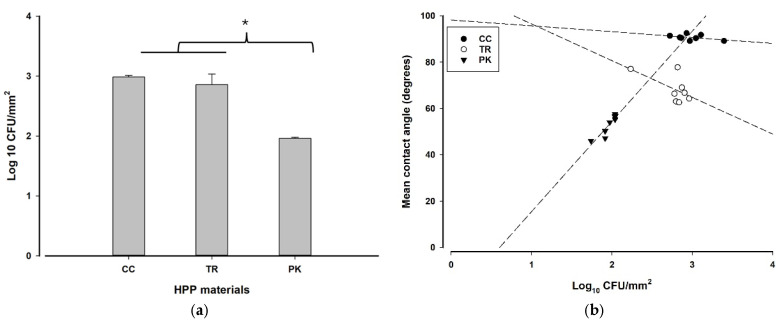
(**a**) Mean and standard deviations of *S. mutans* bacterial counts showing that PK has fewer CFUs than both CC and TR, as indicated by * (*p* < 0.05); (**b**) linear regression analyses of mean contact angles and log_10_ CFU/mm^2^ of the studied materials.

**Figure 4:**
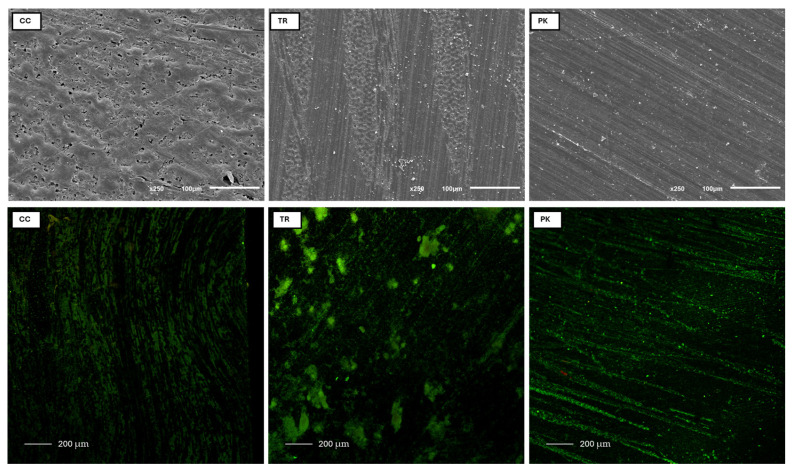
(**Upper**) representative SEM images of CC, TR, and PK specimens (×250 magnification); (**lower**) CLSM of *S. mutans* following the surface patterns on representative specimens, respectively.

The results are presented in Table 2 and illustrated in Figure 2, Figure 3 and Figure 4.

### 3.1. Surface Properties

The average surface roughness measurements (Ra) exhibited significant variations across the three materials, with PK showing the smoothest surface (Ra = 0.161 ± 0.03 µm) in comparison to CC and TR (*p* = 0.0001). A comparable trend was seen across the three materials for the Rz parameter, exhibiting significant differences (*p* = 0.0001). Ra and Rz showed substantial differences in TR specimens (0.359 ± 0.17 µm) than in CC, with the least variation observed in PK (*p* = 0.0001). The average water contact angle measurements varied from 51.36° to 91.03° and were markedly lower in PK, succeeded by TR and CC (*p* = 0.0001). The results demonstrate that PK exhibits greater hydrophilicity compared to FRCs (TR and CC).

### 3.2. Colony-Forming Units

The CFU results showed no statistically significant changes between CC and TR (*p* = 0.377). However, PK revealed markedly reduced *S. mutans* CFU numbers (*p* = 0.000). Furthermore, a strong negative correlation existed between the mean contact angle and CFU for PK (r^2^ = 0.8, *p* = 0.03), but a lesser inverse correlation was observed for CC and TR (Figure 3a,b).

### 3.3. Surface Analysis by SEM and CLSM

The microstructural disparities among the materials are illustrated in the representative SEM images of the HPP material surfaces prior to biofilm formation, as shown in Figure 4, upper. Both CC and TR are reinforced with fibers, yet the configuration of the fibers differs. In CC, the carbon fibers are arranged in a random three-dimensional network, but in TR, the glass fibers are interlaced and more linear across two planes. In contrast, PK is reinforced with fine ceramic filler particles, yielding a smoother surface. The representative CLSM images displayed a bacterial adherence pattern corresponding to the microstructural variations observed on the surfaces of these specimens (Figure 4, lower).

## 4. Discussion

This study highlighted the interplay between the microstructural composition, surface properties, and bacterial adhesion of *S. mutans* on three *high-performance polymeric* (HPP) CAD/CAM composites—CarboCad (CC), TRINIA (TR), and DentoPEEK (PK). The findings revealed that despite the reduced wettability of CC and TR, their CFU levels were significantly greater than those of the relatively hydrophilic PK (*p* = 0.0001). Furthermore, surface roughness exhibited heterogeneity across the materials, with TR displaying the greatest variability, which may affect its bacterial adhesion characteristics, as evidenced by CLSM images. Consequently, both null hypotheses were rejected.

Surface roughness is a pivotal element affecting biofilm development and bacterial adhesion [10]. Ra and Rz are two-dimensional height parameters, with Ra denoting the average surface height and Rz indicating the distance between the highest peak and deepest point on a surface profile. A larger discrepancy between Ra and Rz indicates a rougher surface [25]. This study exhibited the greatest surface roughness in TR, slightly exceeding the clinically acceptable maximum of 0.2 µm for both Ra (0.231 µm) and Rz (0.359 µm). These materials have no antimicrobial properties; however, the differences in their microstructural composition significantly influenced their surface topography, as seen by the SEM images (Figure 4) and confirmed by earlier similar research [20,28]. The layered woven glass fibers in TR produced a more linear fiber configuration, contributing to greater surface irregularities, whereas the random arrangement of carbon fibers in CC exhibited intermediate roughness. PK, conversely, exhibited the smoothest surface owing to its finely homogeneous ceramic-filled composition (0.3–0.5 µm of TiO_2_) comprising 80 wt. % PEEK. These findings align with previous studies that have established the relationship between surface roughness and bacterial adhesion, where rougher surfaces facilitate bacterial attachment and *S. mutans* retention [13,29]. Moreover, the roughness of these FRCs (CC and TR) significantly increased upon aging in three food-simulating liquids (water, 70% ethanol/water, and methyl ethyl ketone) [20] compared to PK. Although rougher restorations were associated with increased plaque retention [30], this correlation was ambiguous for PEEK and other resin-based materials [31,32]. Therefore, more research on these new polymer-based materials should be carried out.

Wettability, determined by mean contact angle measurements, is a crucial component influencing bacterial adhesion. Research shows that materials exhibiting a water contact angle over 62° are classified as highly hydrophobic [33]. In this investigation, CC demonstrated the greatest hydrophobicity, with a mean contact angle of 91.03°, followed by TR at 70.59° and PK at 51.36°. Consequently, both CC and TR materials examined in this research were determined to be hydrophobic, in contrast to PK. Notably, despite their increased hydrophobicity, CC and TR demonstrated substantially higher bacterial adhesion compared to PK. It was previously reported that water-soluble bacteria exhibit a greater affinity for hydrophilic surfaces compared to hydrophobic surfaces [14,15]. However, some studies indicate that enhancing surface hydrophobicity displaces the water barrier between the surface and bacteria, hence promoting closer contact and more robust interactions between them [34,35].

The CLSM images (Figure 4) visually represent the biofilm adhesion outcomes, indicating greater bacterial biofilms surrounding the fibers in CC and TR, whereas PK had a more scattered bacterial distribution, consistent with the surface patterns observed in the SEM images. The increased bacterial adherence on CC and TR is due to their rougher surfaces and fiber-reinforced microstructures, which foster topographies conducive to bacterial colonization [9]. The findings coincide with previous research indicating increased bacterial adherence on rougher and fiber-reinforced surfaces relative to smoother, filler-based materials [16,36]. PK, characterized by its smoother surface and ceramic-filled composition, exhibited less bacterial adherence compared to CC and TR, suggesting it may be a superior material for applications requiring bacterial resistance. Alongside the microstructural variations between the two FRCs and PK, a significant chemical distinction exists between the polymeric materials. CC and TR comprise epoxy resin (40–58 wt. %), whereas PK consists of PEEK (80 wt. %). In comparison to zirconia and chrome–cobalt CAD/CAM framework materials, PEEK was less advantageous due to its comparatively rougher, more hydrophobic surface, resulting in increased susceptibility to microbial adherence [37]. It appears prudent to further examine the susceptibility of various polymers to distinct bacteria.

The results of this study possess significant clinical implications. Increased biofilm formation on these materials may result in plaque accumulation, periodontal inflammation, and secondary caries, especially in high-risk patients or regions of the oral cavity with inadequate hygiene access [38]. Novel fiber-reinforced composites (FRCs), like CC and TR, exhibit advantageous properties and mechanical strength at comparatively low weights [39,40]; however, this work demonstrated their vulnerability to bacterial adherence, which may jeopardize their long-term clinical efficacy. Consequently, selective clinical application is essential where full coverage from oral fluids appears advisable.

Despite the beneficial insights provided in this study, several drawbacks should be acknowledged. The in vitro design of this study fails to completely imitate the complex and dynamic conditions of the oral environment, where variables such as saliva flow, oral fluids, and multi-species microbial interactions significantly influence biofilm development. Future studies should integrate these variables to more accurately replicate clinical scenarios. This research concentrated exclusively on *S. mutans*, a singular bacterial species, although in vivo biofilms comprise varied microbial populations. Investigating the adhesion of additional clinically significant pathogens, such as *Porphyromonas gingivalis* and *Candida albicans*, might yield a more thorough comprehension of bacterial interactions with these materials. Furthermore, this work examined the early phases of biofilm formation; however, longitudinal research is required to assess the impact of aging, wear, and recurrent exposure to oral fluids on the surface characteristics and bacterial adhesion of these materials. Future research must concentrate on developing innovative modifications to the surface and conducting extensive clinical investigations to corroborate these findings and enhance the in vivo performance of HPP composites. For example, surface modifications or seal coatings may be engineered to diminish bacterial adhesion on fiber-reinforced composites while maintaining their mechanical strength. Antibacterial coatings, including those with silver nanoparticles, have demonstrated efficacy in diminishing biofilm formation on dental materials [41,42]. Moreover, optimizing the polishing methods to reduce surface roughness may improve the resistance of fiber-reinforced composites to bacterial adherence [43,44]. To conclude, this work highlighted the multifactorial aspects of bacterial adherence, indicating that surface roughness, wettability, and microstructural composition of the material interact to influence biofilm formation. This underscores the necessity of monitoring all these aspects comprehensively when assessing the performance of dental restorative materials and striving to enhance their clinical outcomes, including less bacterial adhesion.

## 5. Conclusions

The fiber-reinforced CAD/CAM composites (CC and TR), despite their high hydrophobicity and roughness, exhibited increased susceptibility to bacterial adhesion compared to the smoother and relatively hydrophilic ceramic-filled PEEK (PK). This finding emphasized the complex influence of their variations in microstructural composition and surface topography. Subsequent research should focus on improving the surface finish of these *high-performance polymeric* composites prior to broadening their clinical applications.

## Figures and Tables

**Table 1:** *High-performance polymer* (HPP) CAD/CAM composites and their manufacturer information.

CAD/CAM Material	Code	Manufacturer
Carbon fiber-reinforced composite	CC	CarboCAD 3D Dream frame, DEI^®^italia, Torino, Italy
Glass fiber-reinforced composite	TR	TRINIA, Bicon Europe, Ltd., Limerick, Ireland
Ceramic-filled polyether ether ketone	PK	DENTOKEEP, NT-Trading, Karlsruhe, Germany

**Table 2:** The means and standard deviations of roughness (Ra and Rz), water contact angle measurements, and count of adhering *S. mutans* to the surface of three HPP composites (CC, TR, and PK).

Measurements	CC	TR	PK
Ra (µm)	0.194 (0.03) ^a^	0.231 (0.06) ^b^	0.161 (0.03) ^c^
Rz (µm)	0.107 (0.03) ^a^	0.359 (0.17) ^b^	0.055 (0.02) ^c^
Contact angle (°)	91.03 (1.10) ^a^	70.59 (5.45) ^b^	51.36 (3.98) ^c^
*S. mutans* (Log_10_ CFU/mm^2^)	2.986 (0.03) ^a^	2.859 (0.17) ^a^	1.963 (0.02) ^b^

For each row, different *superscript lowercase letters* present statistically significant differences between groups (Dunnett T3, *p* ≤ 0.05).

## Data Availability

All data analyzed during this study are included in this published article. Additionally, the complete dataset supporting the conclusions of this article is available from the corresponding author and can be accessed upon reasonable request.

## Abbreviations

The following abbreviations are used in this manuscript:

HPP	High-performance polymer
CAD/CAM	Computer-aided design/computer-aided manufacturing
CFU	Colony-forming unit
CLSM	Confocal laser scanning microscope
